# Supplementary figures and images for: Modulation of host lipid metabolism by virus infection leads to exoskeleton damage in shrimp
Source: PLoS Pathog. 2024 May 13;20(5):e1012228. doi: 10.1371/journal.ppat.1012228 (PMC11115362; doi:10.1371/journal.ppat.1012228)

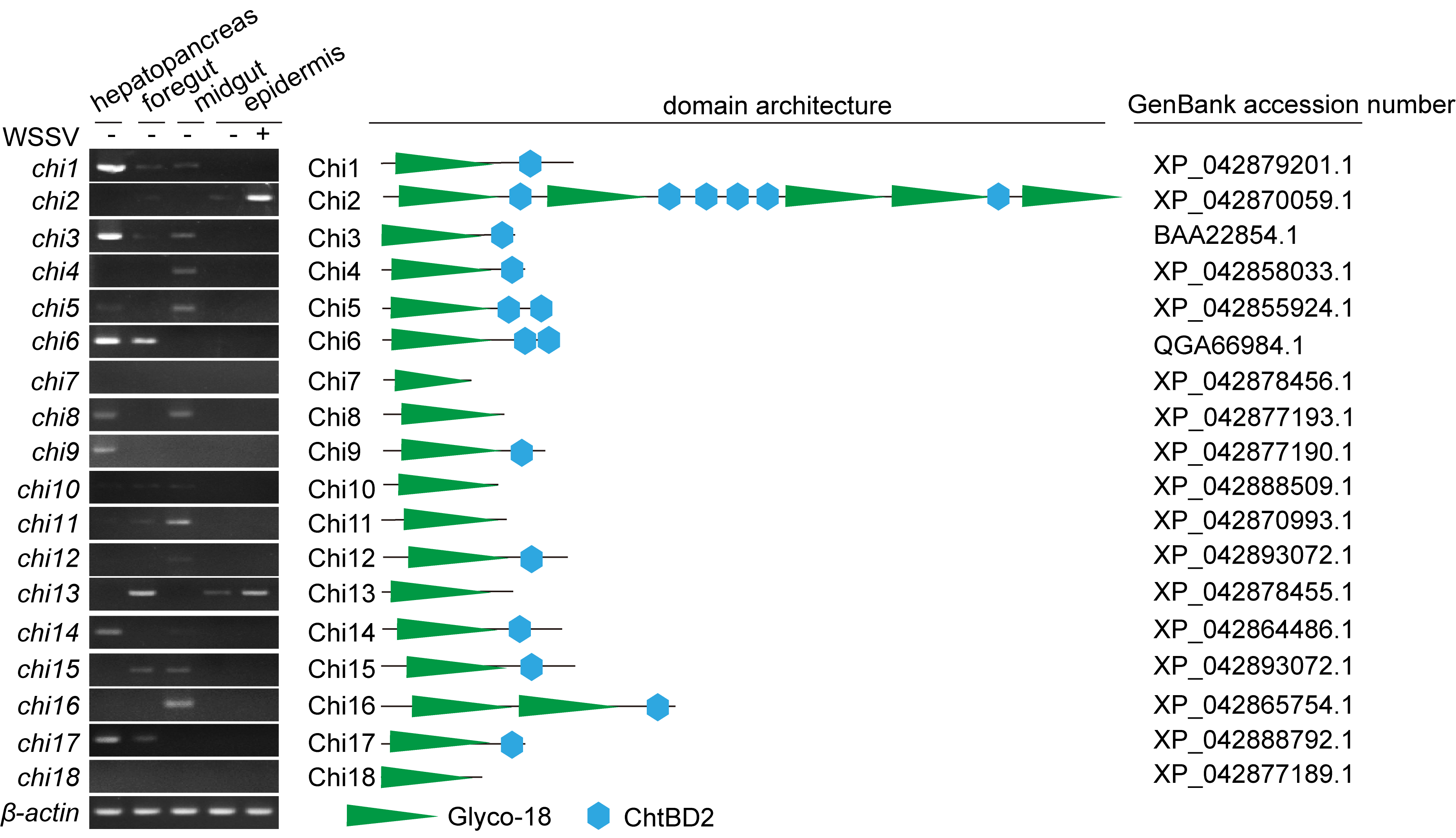

Supplement: S1 Fig — Left, tissue distribution and expression changes after WSV infection of shrimp chitinases, as analyzed using RT-PCR. The images are representative of three independent replicates. Middle, domain architecture of the chitinase family analyzed using the online tool SMART (smart.embl.de). Right, accession numbers for the chitinase sequences. (TIF) [file ppat.1012228.s001.tif]

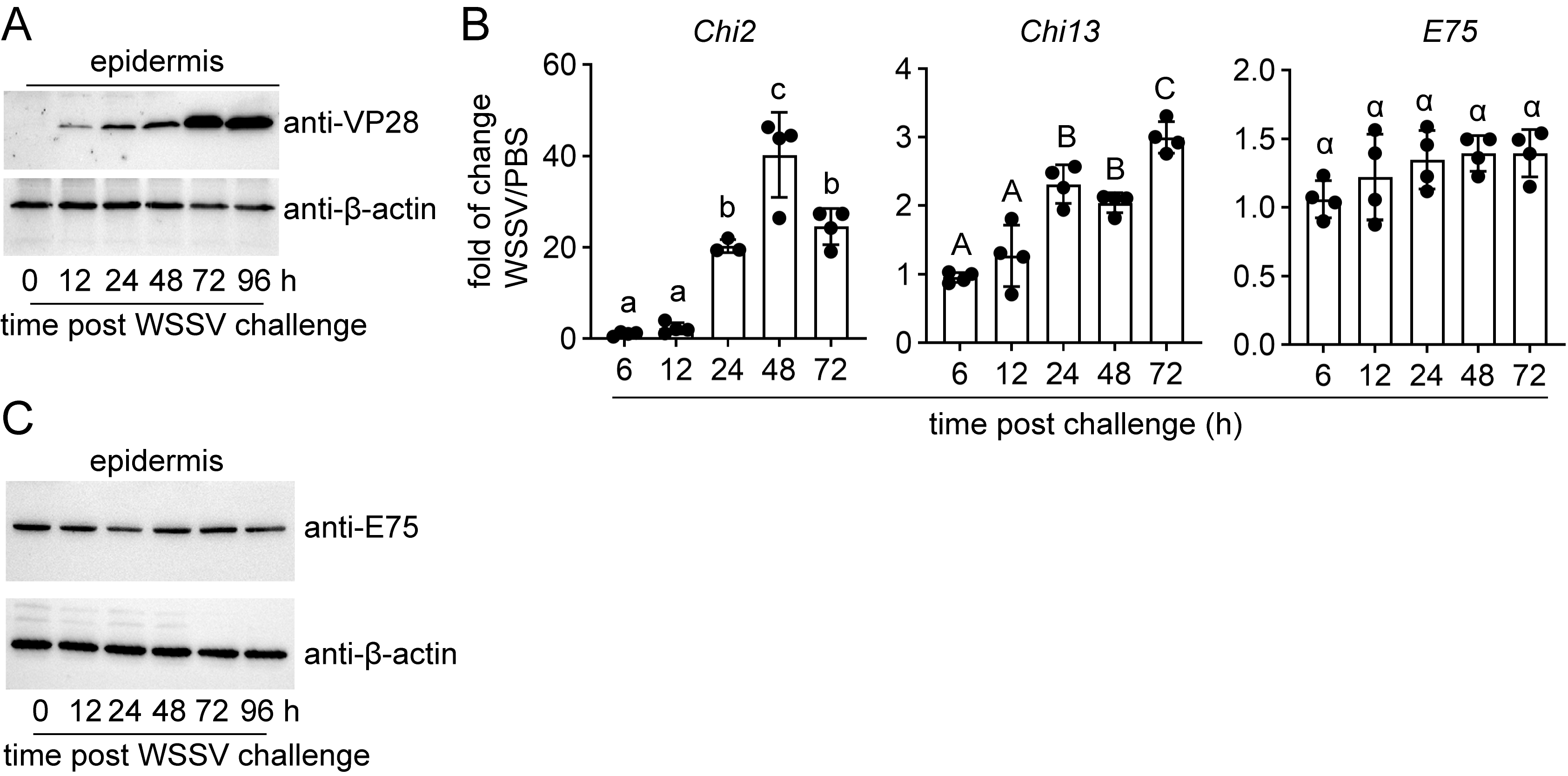

Supplement: S2 Fig — (A) WSSV VP28 level in the sub-cuticle epidermis. Shrimp were injected with a WSSV inoculum, and VP28 levels were analyzed using western blotting with β-actin as the internal reference. The blot images are representative of three independent replicates. (B) Temporal expression profiles of Chi2, Chi13, and E75 in the epidermis after WSSV infection analyzed by qRT-PCR with β-actin as the internal reference. The fold change in expression levels in the WSSV group compared with the PBS group at each time point is shown. Mean ± SD of four independent replicates. Different characters indicate significant differences, analyzed by one-way ANOVA. (C) E75 level in the sub-cuticle epidermis. Shrimp were injected with a WSSV inoculum, and E75 levels were analyzed using western blotting with β-actin as the internal reference. Images of blots are representative of three independent replicates. (TIF) [file ppat.1012228.s002.tif]

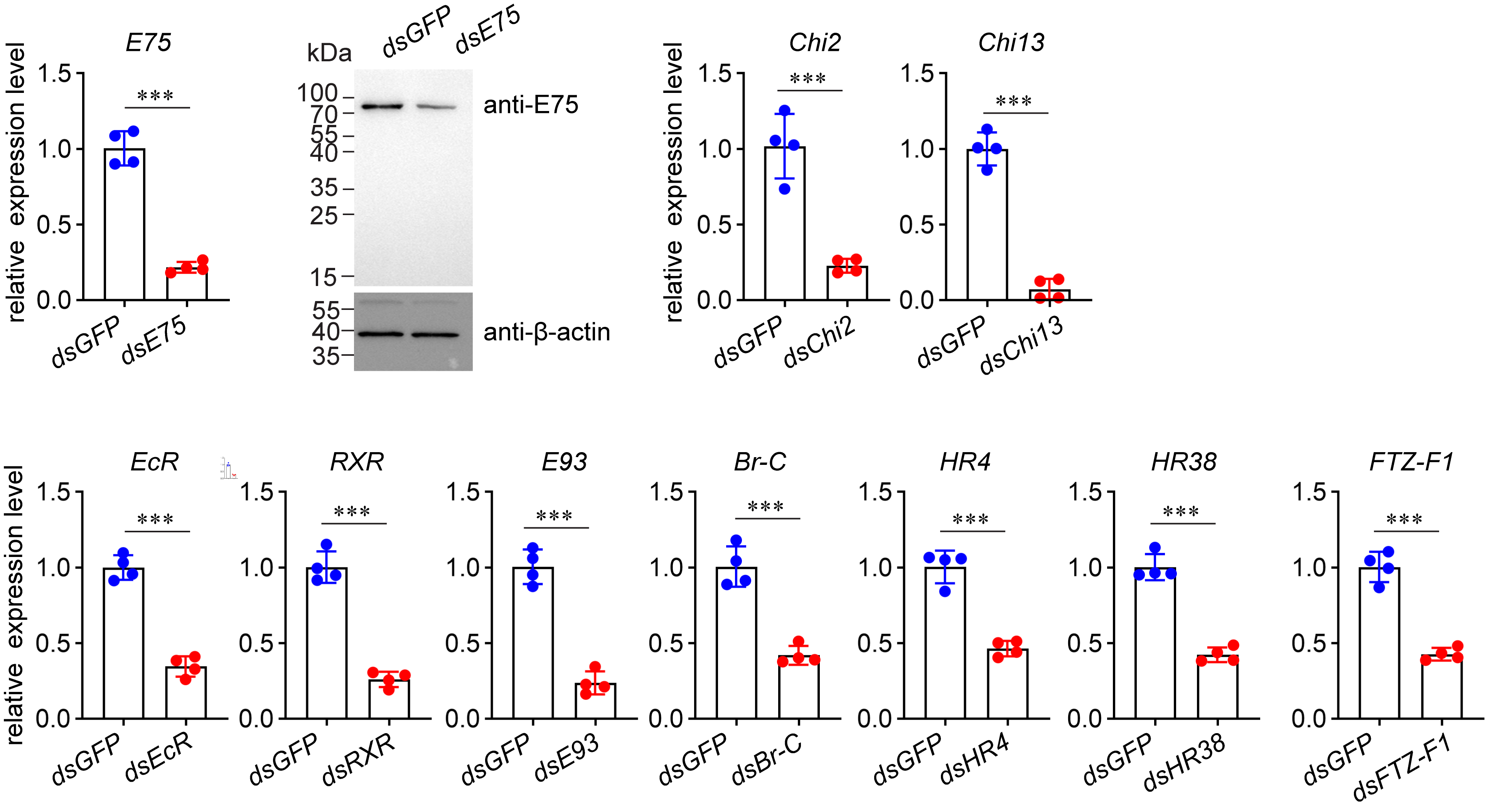

Supplement: S3 Fig — Indicated dsRNAs at a dose of 5 μg/g of shrimp were injected into shrimp hemocoels. The knockdown efficiency in the sub-cuticle epidermis was determined by using qPCR at 24 h after dsRNA application. Mean ± SD from four independent replicates, unpaired Student’s t-test, ***p < 0.001. E75 levels were also analyzed using western blotting with β-actin as the internal reference. Images of blots are representative of three independent replicates. (TIF) [file ppat.1012228.s003.tif]

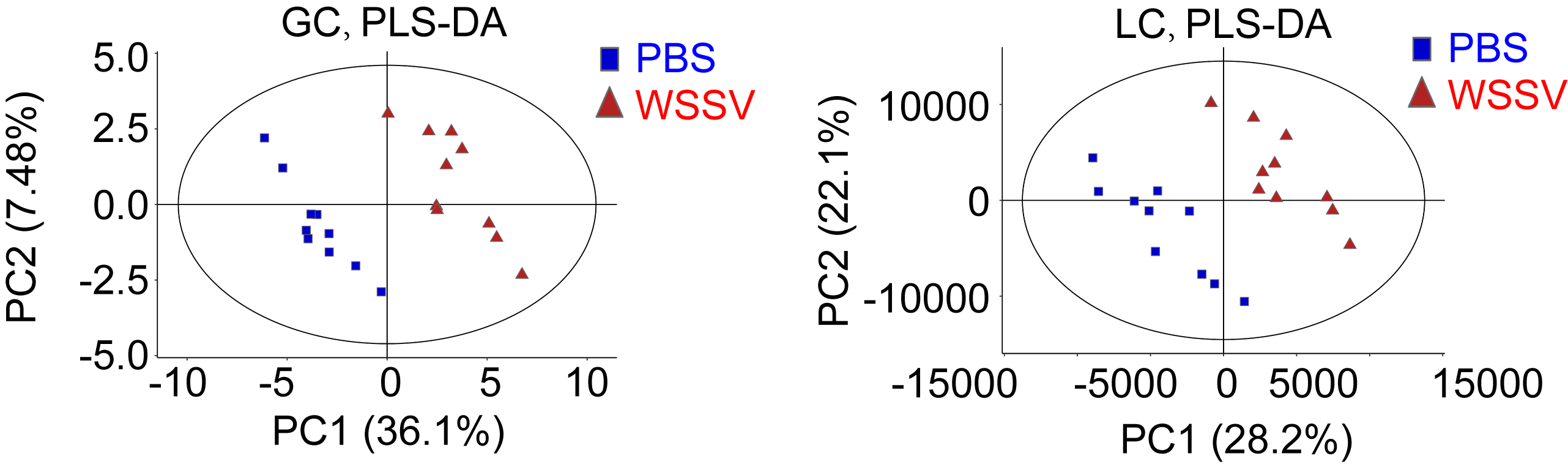

Supplement: S4 Fig — (TIF) [file ppat.1012228.s004.tif]

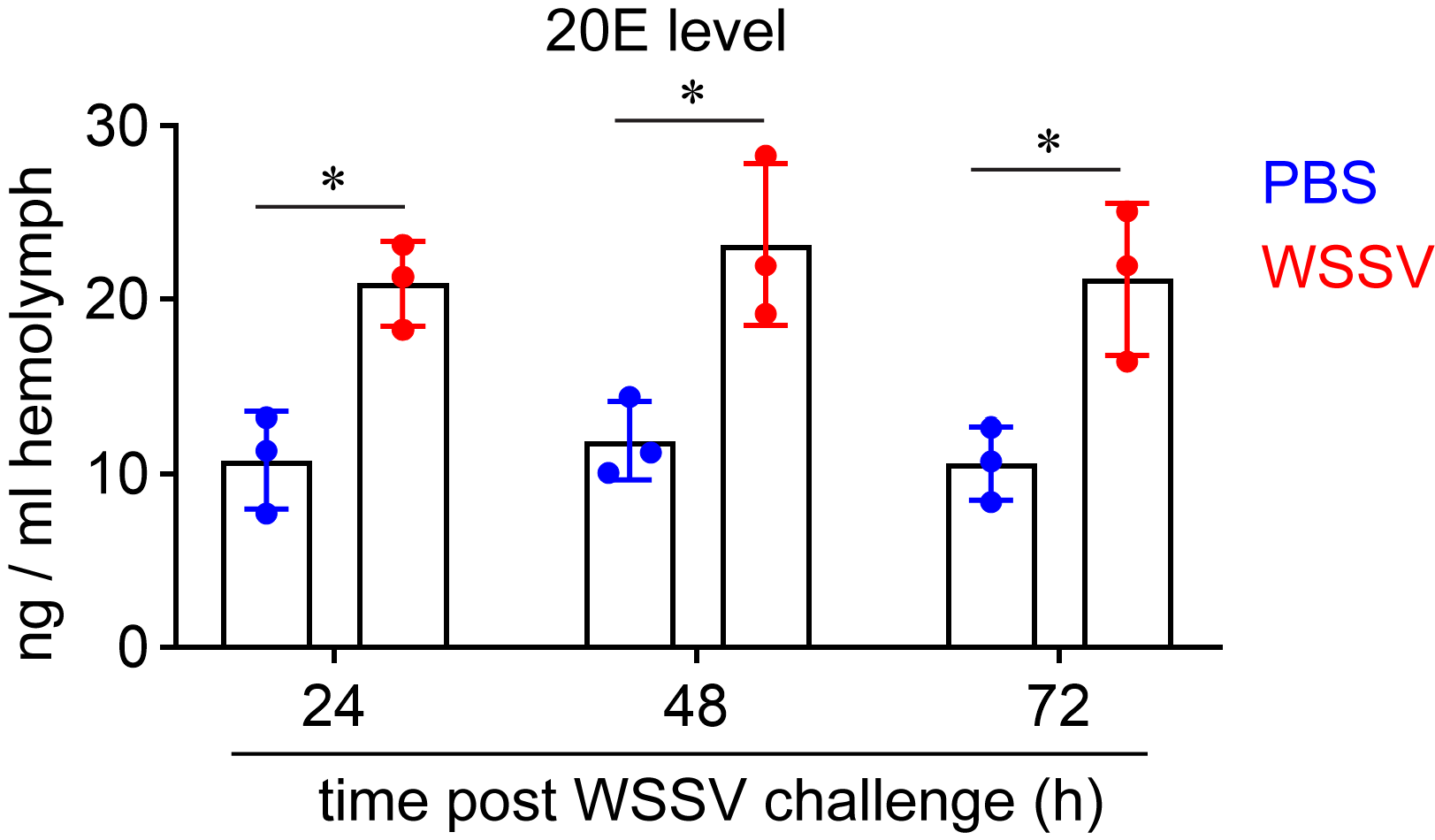

Supplement: S5 Fig — 20E concentration was determined using a 20E ELISA kit (Mlbio, Shanghai, China; ML521987) according to the manufacturer’s instructions. Absorbance at 450 nm was monitored using a Multiskan FC microplate reader (Thermo Fisher Scientific, Waltham, MA, USA). The amount of 20E was determined using a standard curve generated from a gradient dilution of the standard samples. (TIF) [file ppat.1012228.s005.tif]
