# Supplementary material for: Modulation of host lipid metabolism by virus infection leads to exoskeleton damage in shrimp
Source: PLoS Pathog. 2024 May 13;20(5):e1012228. doi: 10.1371/journal.ppat.1012228 (PMC11115362; doi:10.1371/journal.ppat.1012228)
Supplement: S1 Video — Shrimp was transferred into a new glass tank containing fresh seawater at 72 h after WSSV infection or 48 h after MA injection, and was allowed to acclimatize for 30 min. A glass rod was used to prod the shrimp lightly, and the video was recorded. (PPTX) [file ppat.1012228.s006.pptx]

## Slide 1
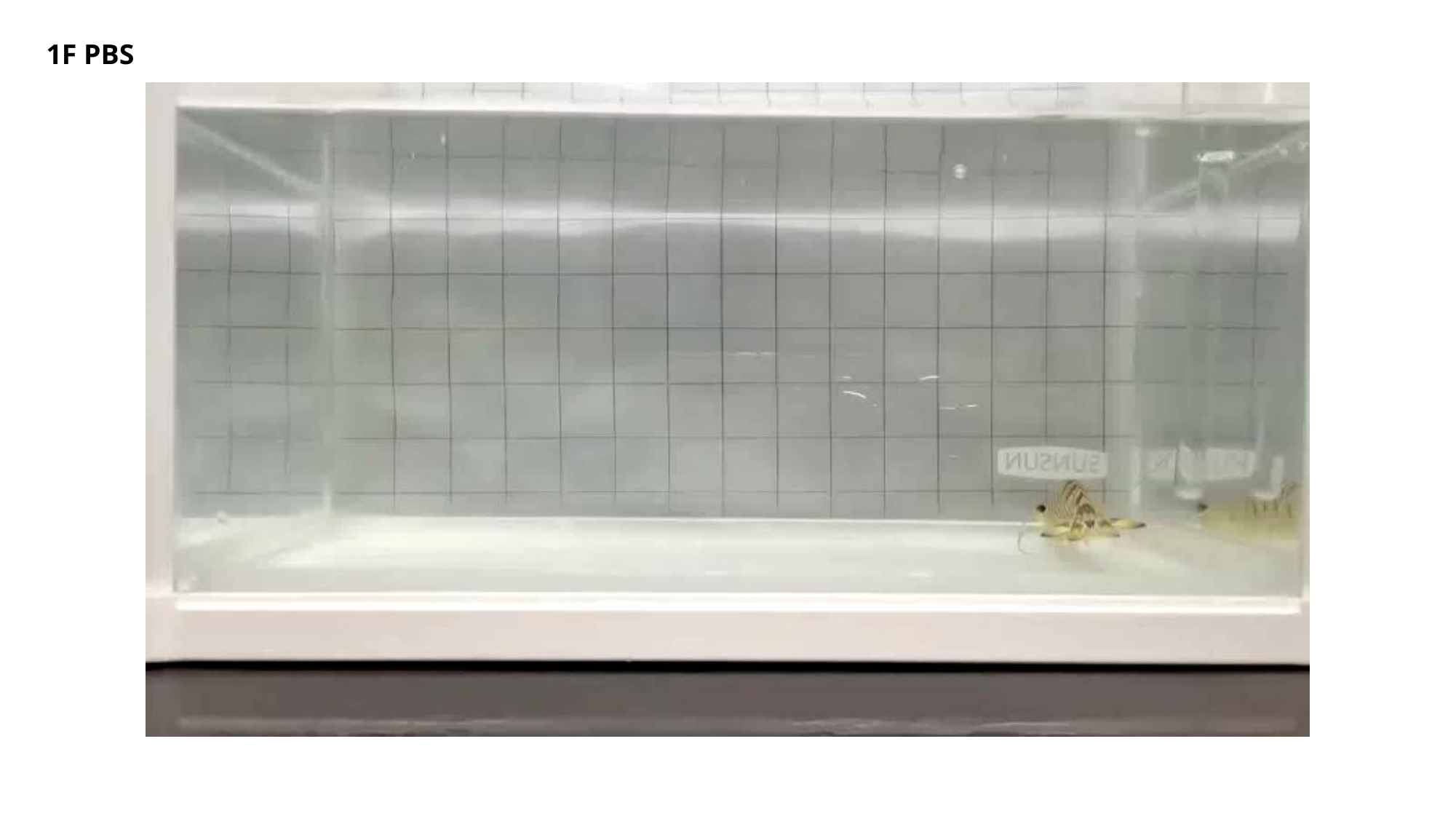

1F PBS

## Slide 2
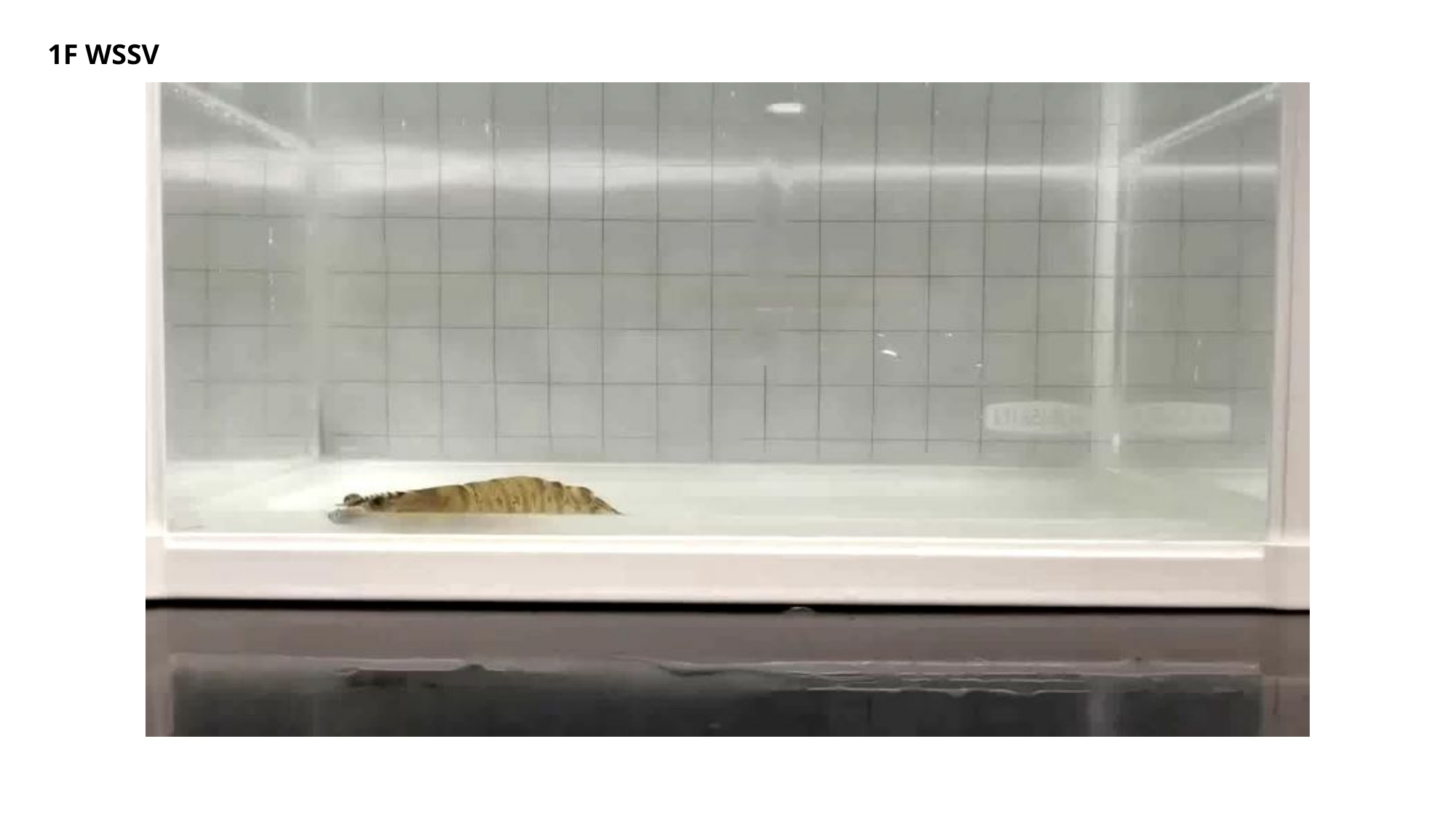

1F WSSV

## Slide 3
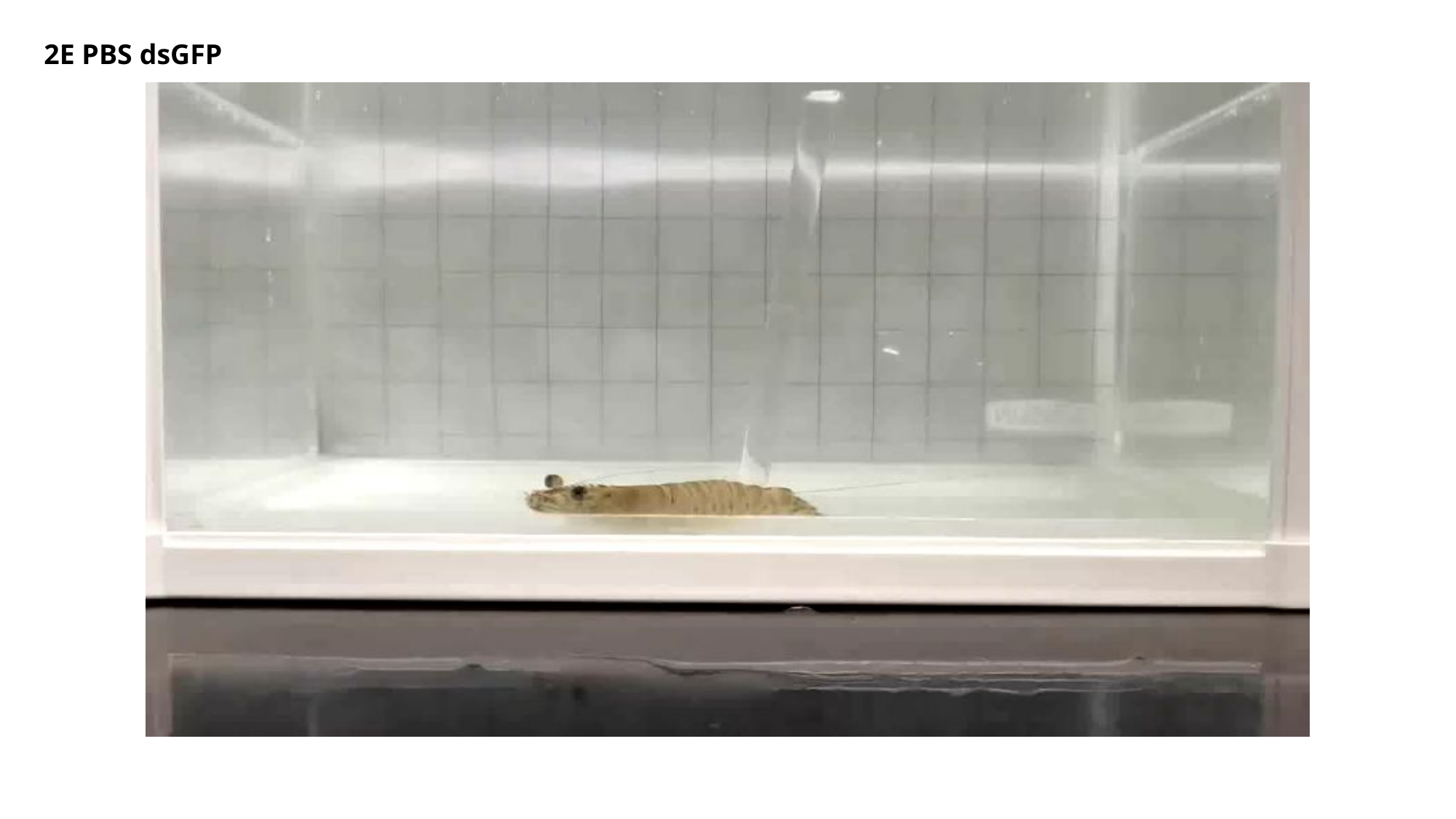

2E PBS dsGFP

## Slide 4
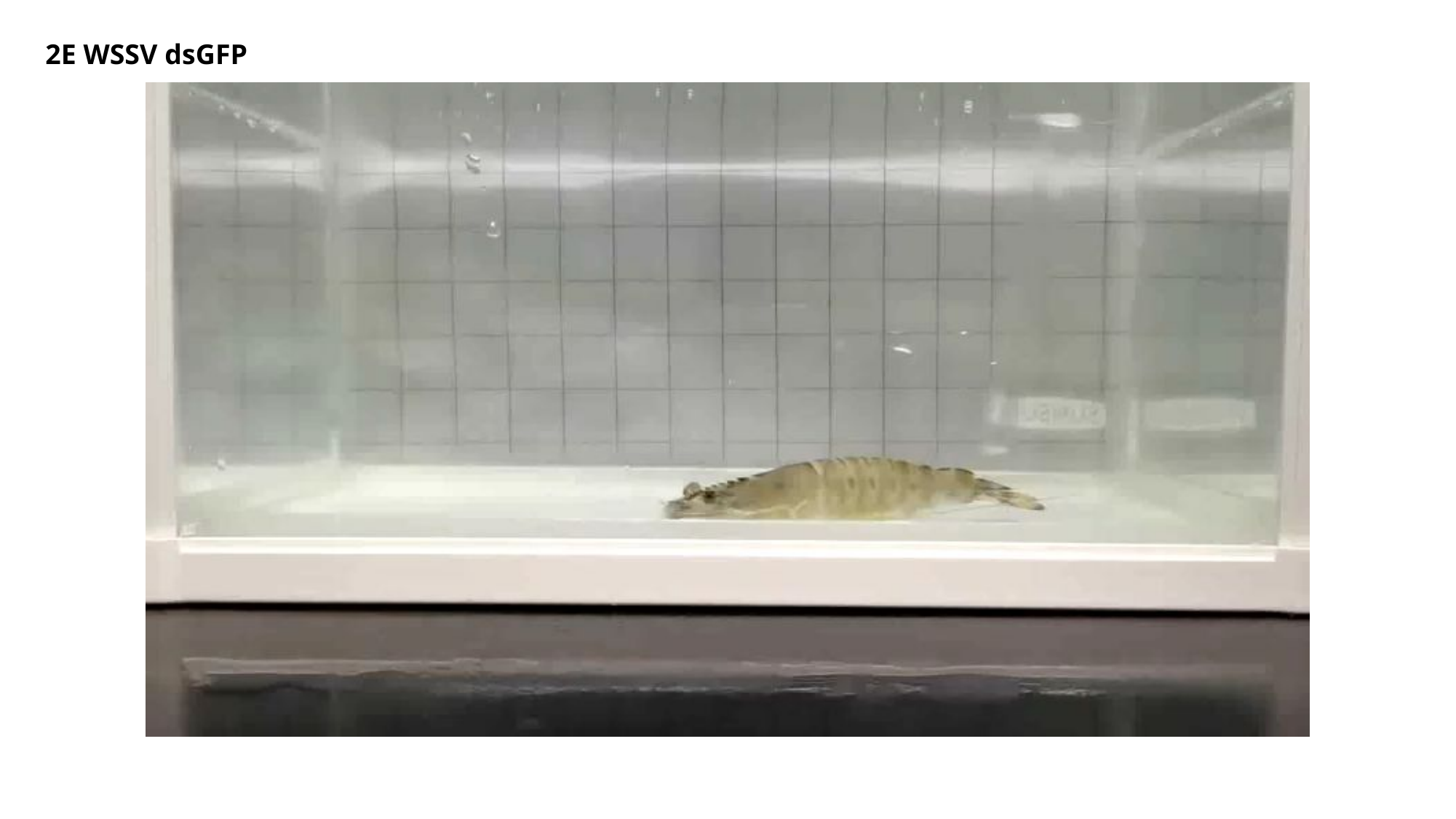

2E WSSV dsGFP

## Slide 5
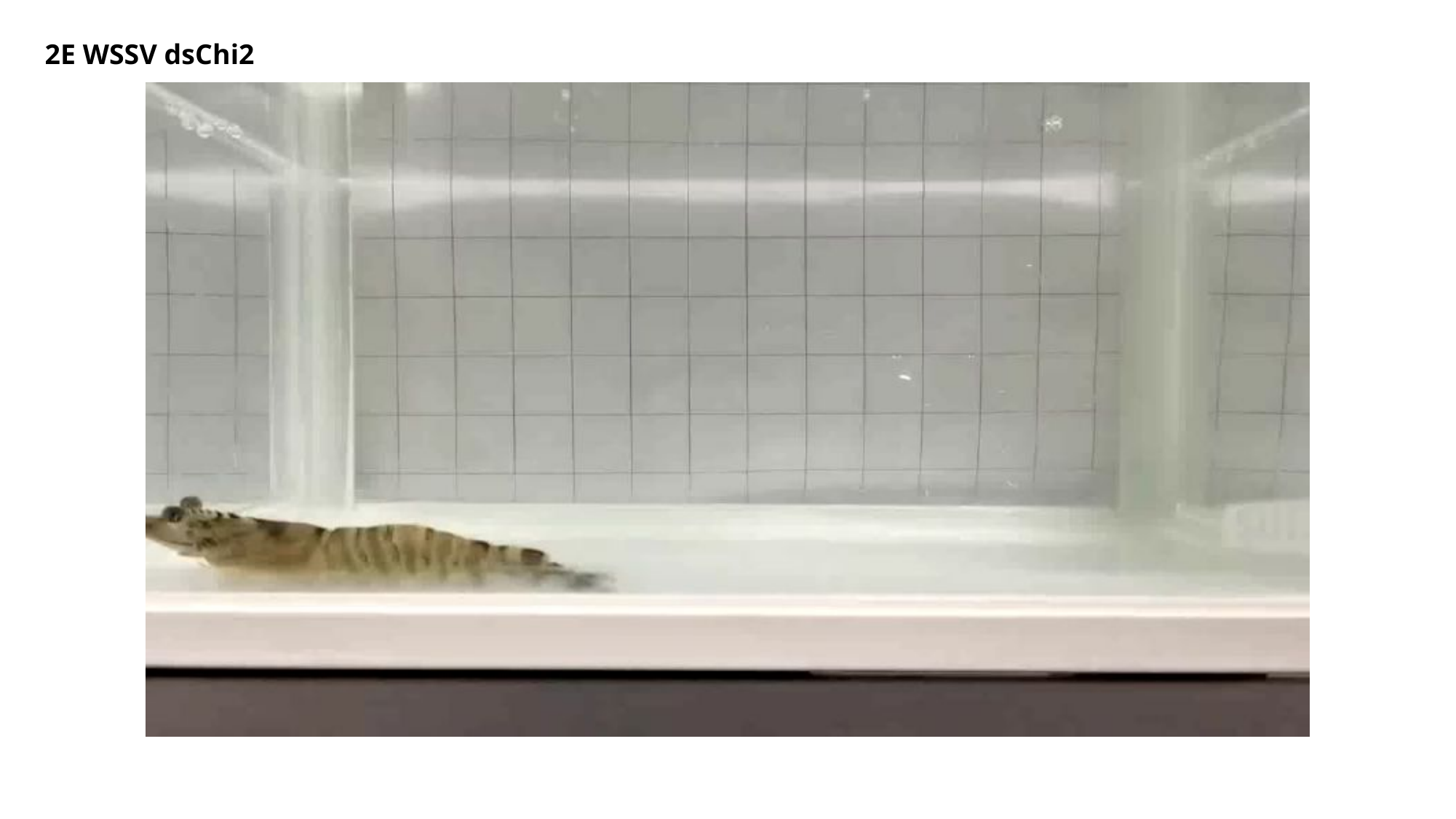

2E WSSV dsChi2

## Slide 6
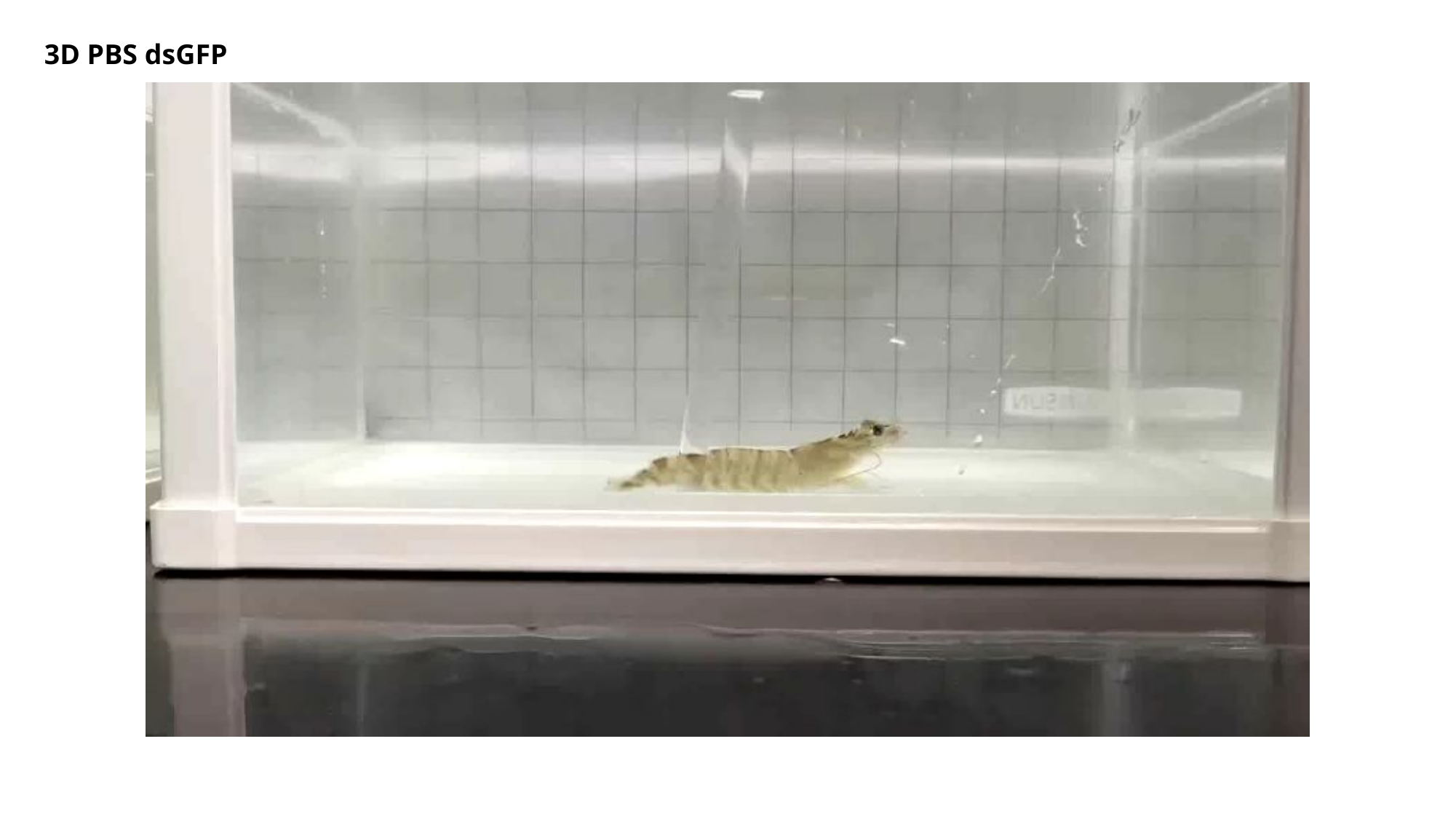

3D PBS dsGFP

## Slide 7
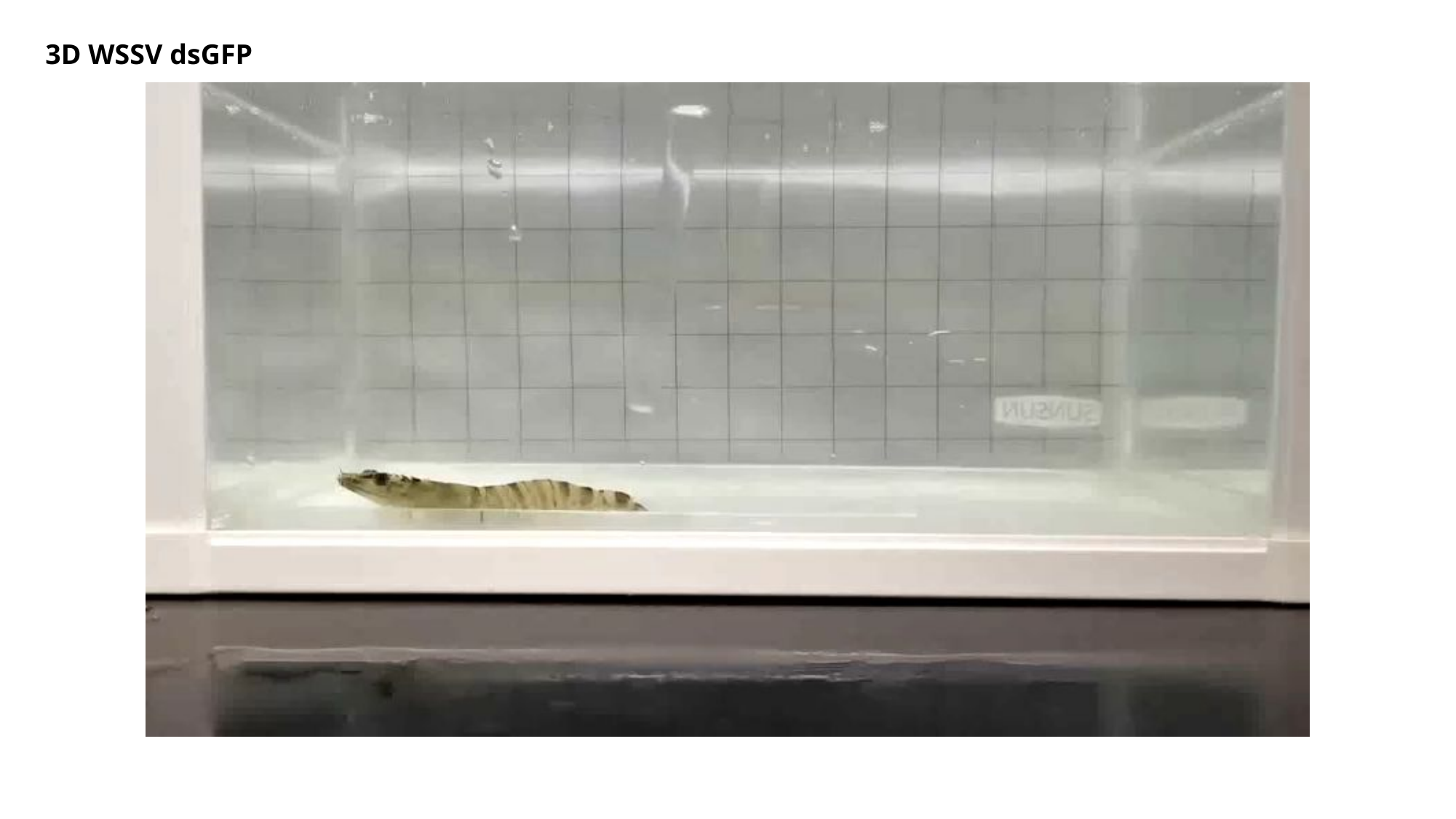

3D WSSV dsGFP

## Slide 8
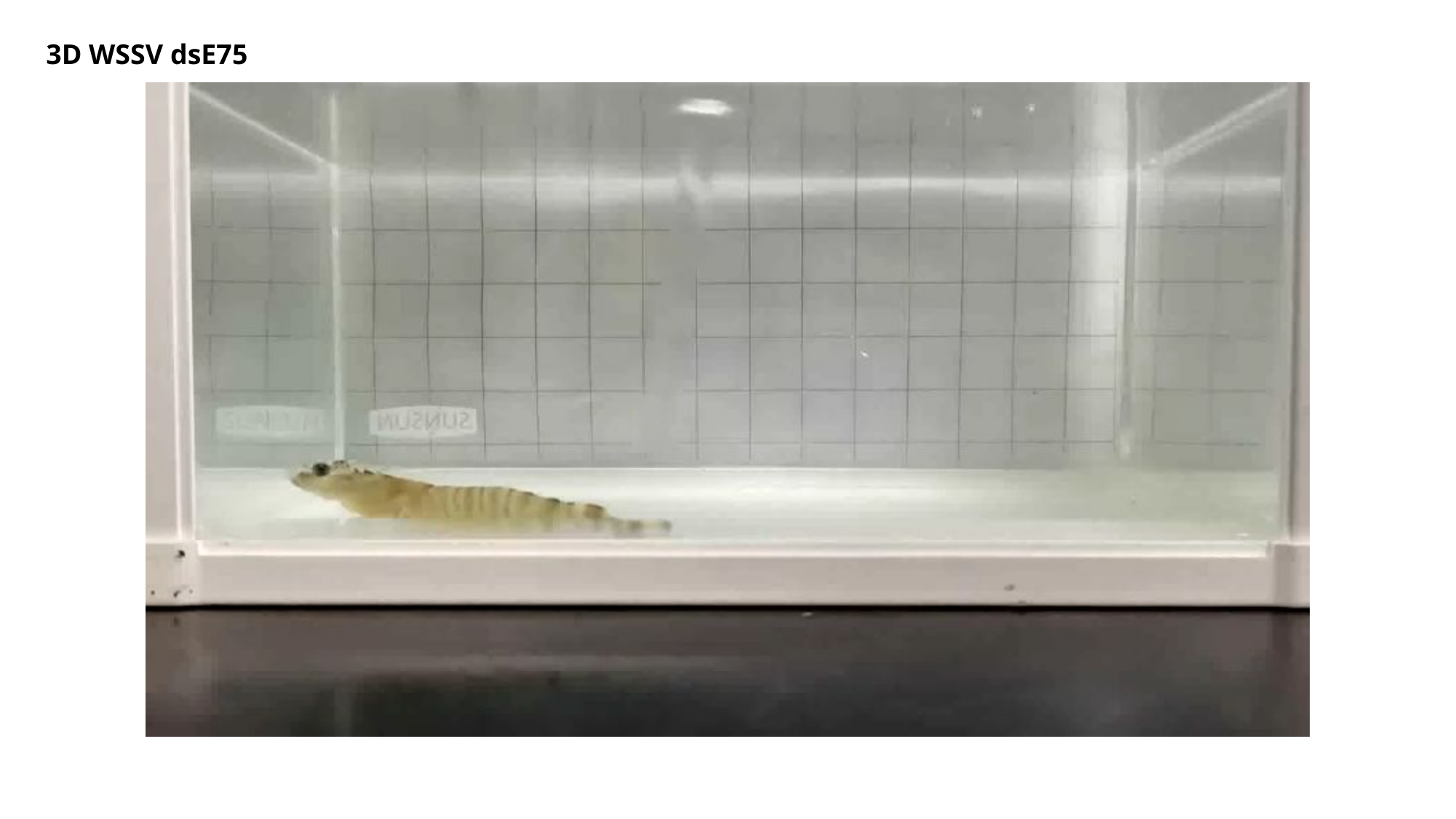

3D WSSV dsE75

## Slide 9
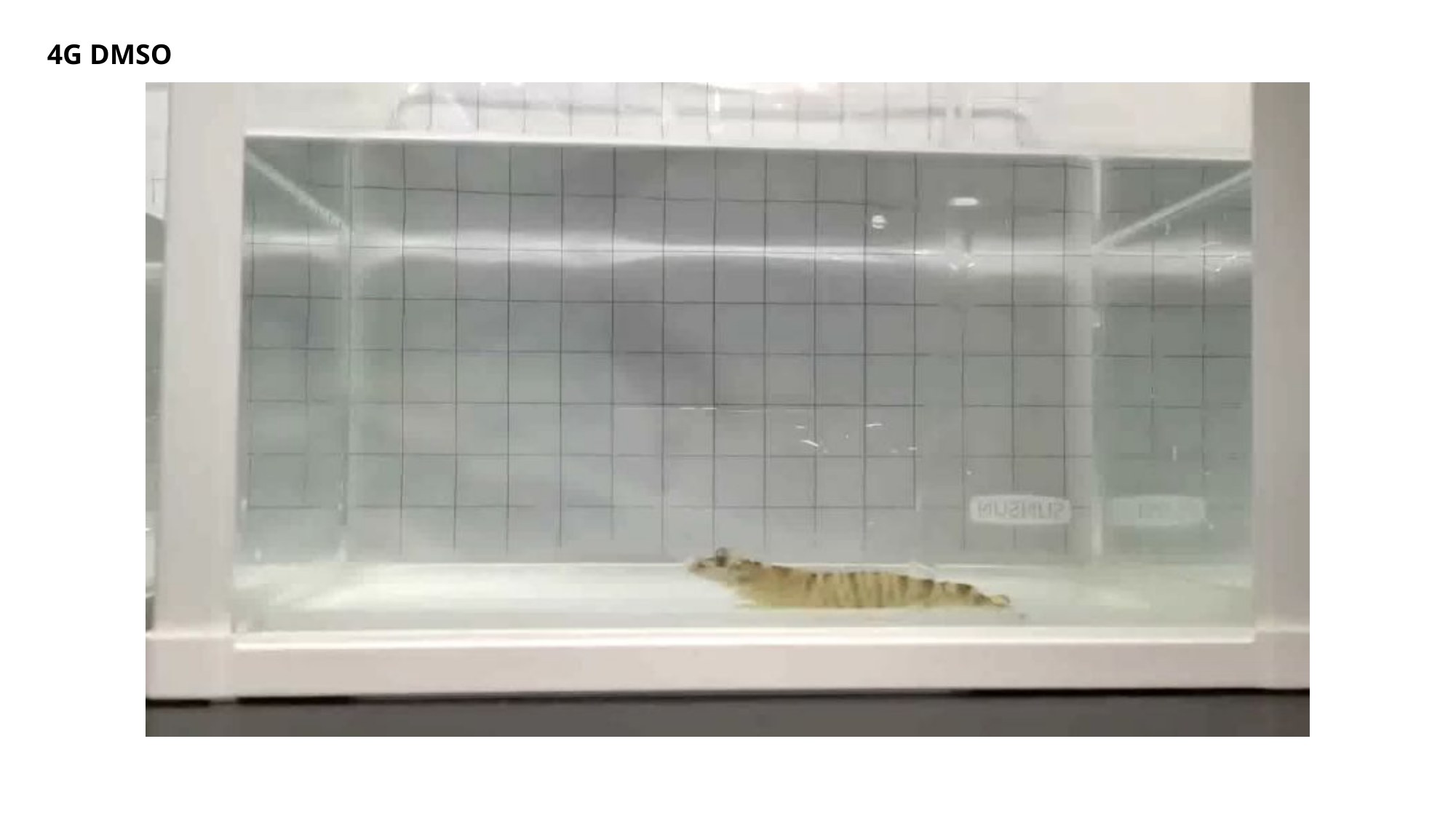

4G DMSO

## Slide 10
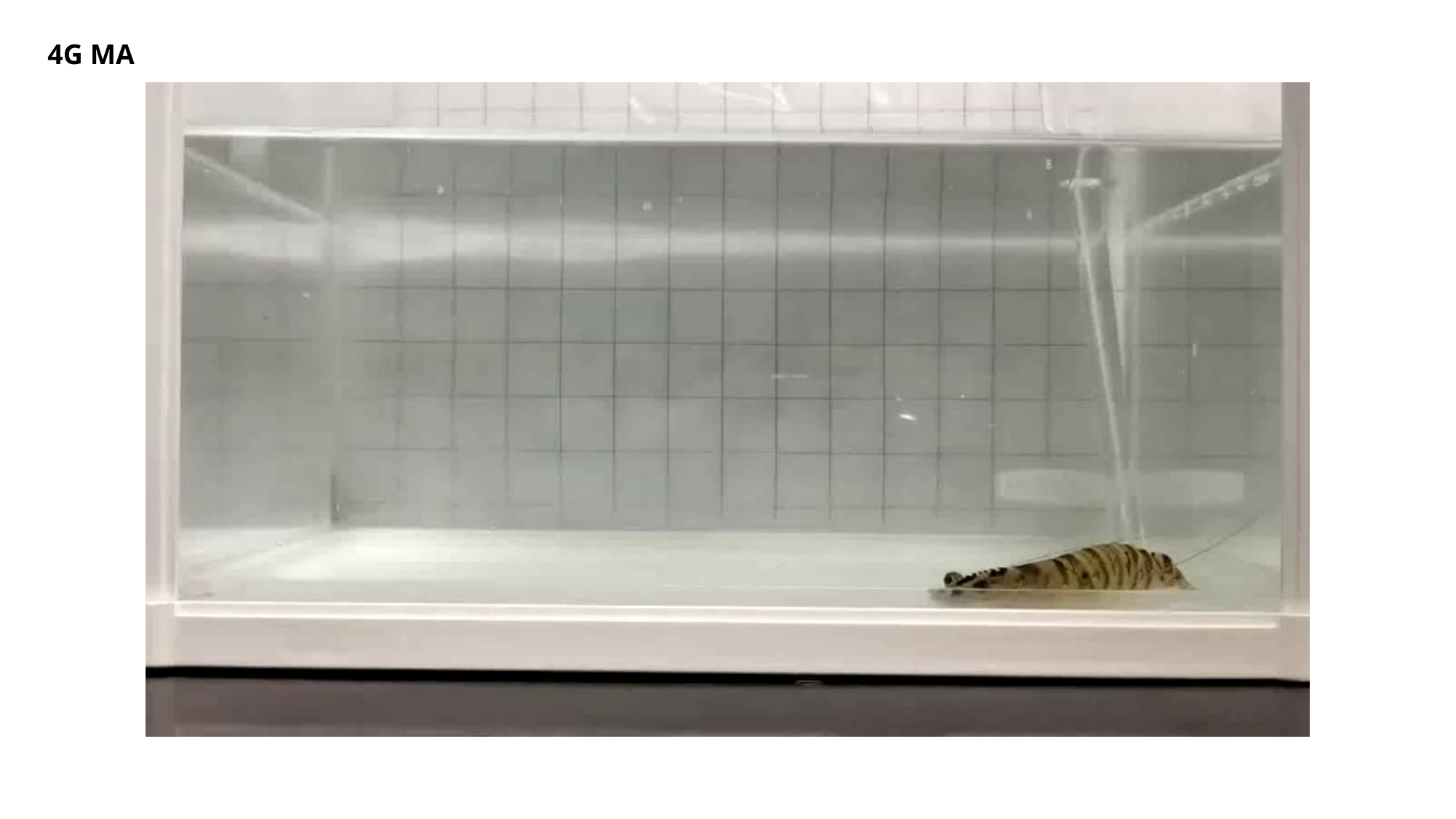

4G MA

## Slide 11
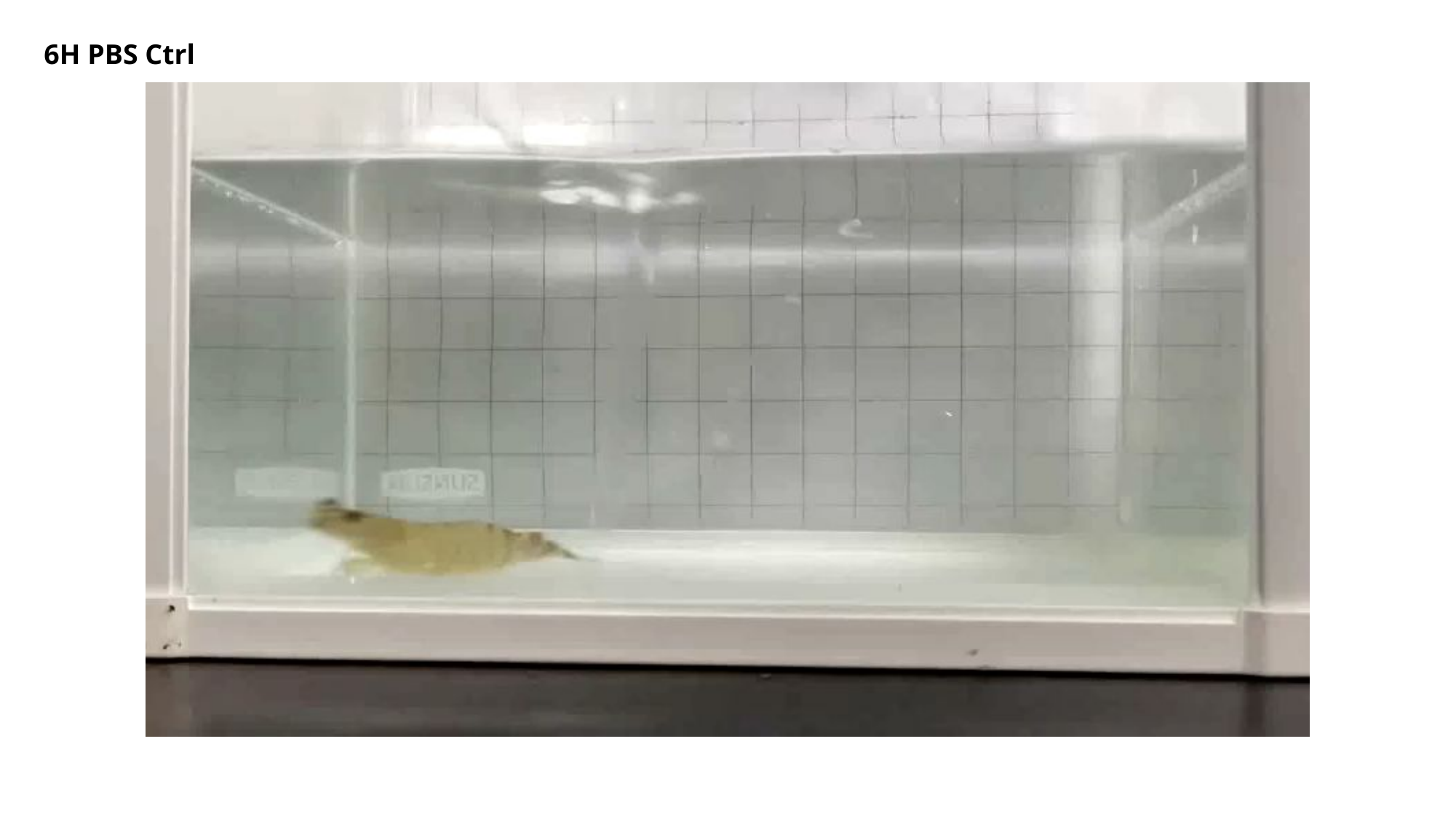

6H PBS Ctrl

## Slide 12
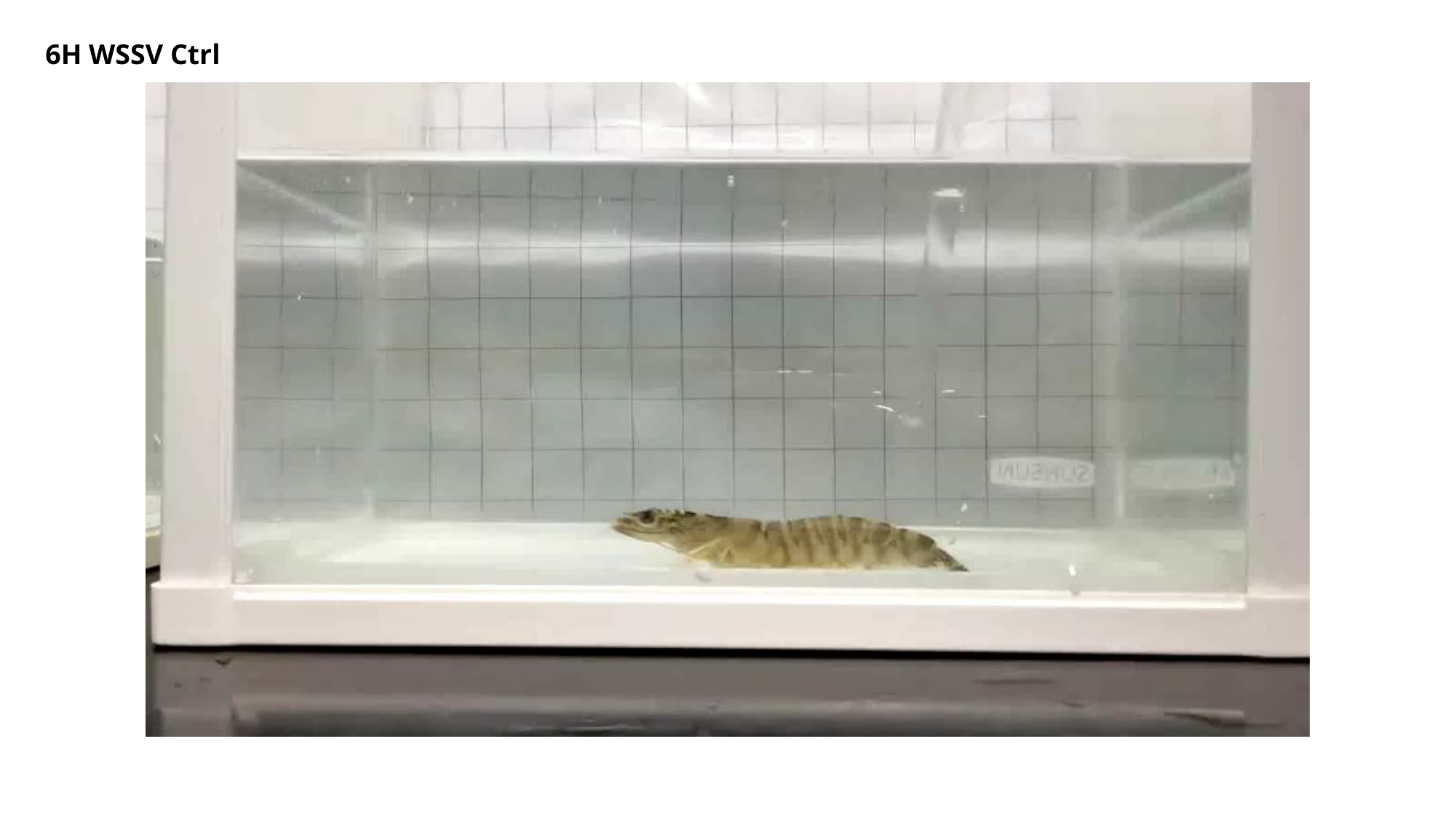

6H WSSV Ctrl

## Slide 13
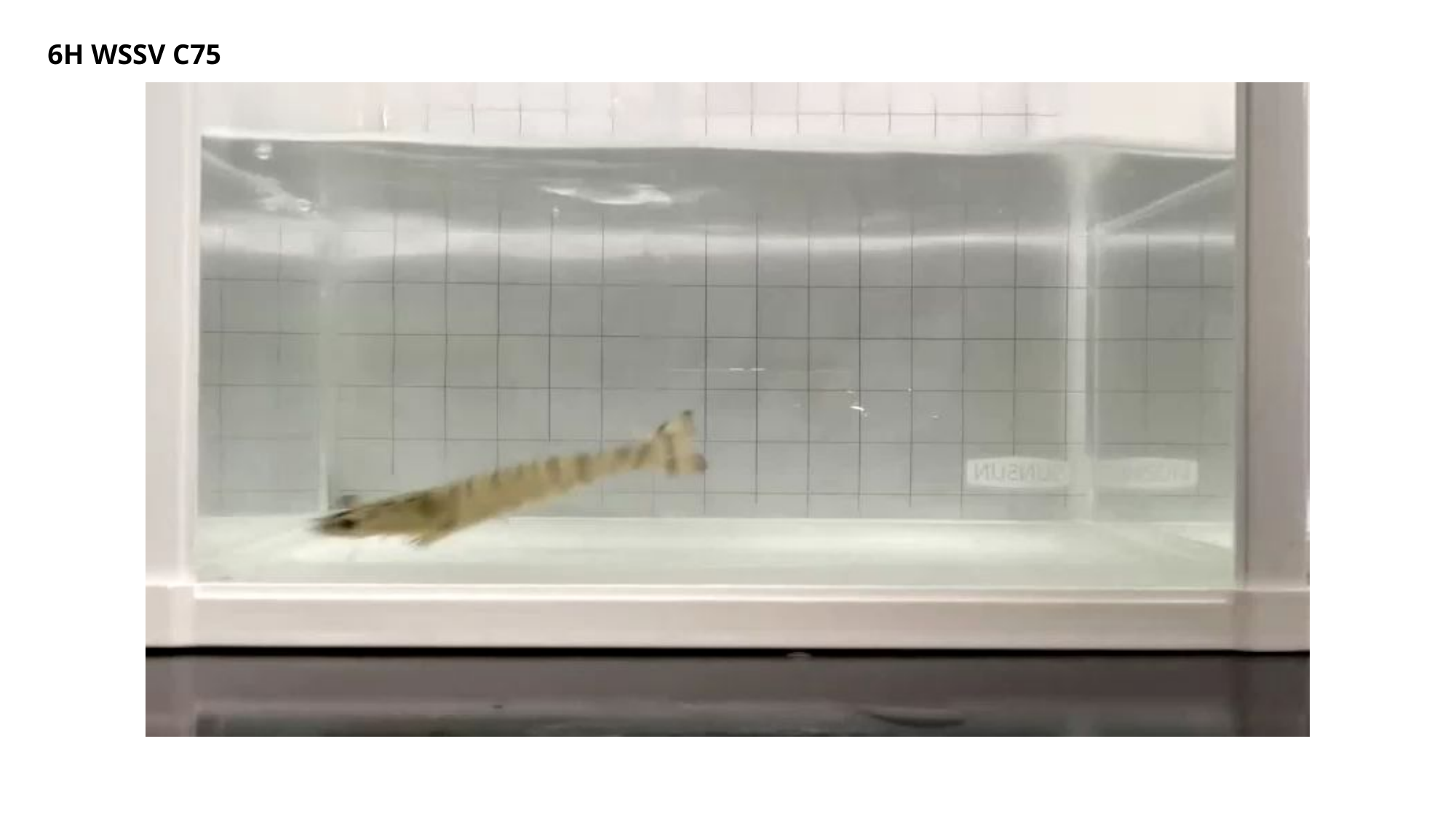

6H WSSV C75
