# Supplementary material for: Modulation of host lipid metabolism by virus infection leads to exoskeleton damage in shrimp
Source: PLoS Pathog. 2024 May 13;20(5):e1012228. doi: 10.1371/journal.ppat.1012228 (PMC11115362; doi:10.1371/journal.ppat.1012228)
Supplement: S1 Table — (DOCX) [file ppat.1012228.s007.docx]

| Primers | Sequence (5′–3′) |
| --- | --- |
| **RT-PCR** |  |
| Chi1 RTF | GTTGGCTGACCCGAGAAT |
| Chi1 RTR | GCCGATGAACGAGTAGATGA |
| Chi2 RTF | TCACCCCGATGACGAAAAC |
| Chi2 RTR | CCGCTGTAGGCATAAGGACC |
| Chi3 RTF  Chi3 RTR  Chi4 RTF  Chi4 RTR  Chi5 RTF  Chi5 RTR  Ch6 RTF  Chi6 RTR  Chi7 RTF  Chi7 RTR  Chi8 RTF  Chi8 RTR  Chi9 RTF  Chi9 RTR  Chi10 RTF  Chi10 RTR  Chi11 RTF  Chi11 RTR  Chi12 RTF  Chi12 RTR  Chi13 RTF  Chi13 RTR  Ch14 RTF  Ch14 RTR  Ch15 RTF  Ch15 RTR  Chi16 RTF  Chi16 RTR  Chi17 RTF  Chi17 RTR  Chi18 RTF  Chi18 RTR | ACCGCAAGTACCACCTCAT  AGCCCAATCGCAGTAGAAG  CAACCCGCTCTTGTCTGTC  GCACTCGCCTTTGTTCACC  AGGAGCAGACAGGAAGAAGC  ATAGCCGAGAATCCCCAGA  ATACCTGGGACTCTCGGGG  ATTAGGGCGGAAATCATCG  CTTATGCCCAGCAACTCG  ATACGCTACCATTTCCAGG  AGTGGAAGAGGGAATGGG  TTTGCTCCTTGGCACAGTA  AAGATGGACTTCGTGCGGG  TGGTTGTGGGAGCGTTGG  TCATCGGGATTCCTACTTAT  CTTTTGCCCACTTTACCTT  ACATCCACCTTTCCTCCTG  CTTTGGGCATTCCCTTTT  CGCCACATCCTACCAACCT  GACCAAACCATCGCTCCAG  AGGCTTCTACGGCAGGAC  TGTGAGGGCTTTGTTGATT  CGACCCCAACAACAATGAC  CCTCGTAGCCAACCCACT  GCTTTCTTTCCGCCCTC  GCTCCTTTCCCATAGTTGAT  AGATGCTCGGAATCCCTCG  GGACCACGAGGTGTAGTAGC  GCAGCCAGCCAGGTCTCA  GCACATTCGCTTAGCAGTCATT  GGTTACGATGTCCCAGAGA  GATGTTAGCGAGGGTGAAG |
| EcR RTF | GCAAAGGACCTCAACCAGTA |
| EcR RTR  RxR RTF  RxR RTR  FTZ RTF  FTZ RTR  E75 RTF  E75 RTR  Br-C RTF  Br-C RTR  E93 RTF  E93 RTR  HR4 RTF  HR4 RTR | CAGAGTCGTCGTCAGTTATTC  GAAGGGAGATAAAGAGGTGG  AGGAGGCGATTAGCAGTT  GAGTCCTTTCCTCTCTTCGTC  GATGATGTGCGGGATTCG  CGTCTGAATCTCCTCTGA  CACTCTTCTCGTCAATGG  TAAGTGAAGCCAGGGACAG  CAGACTCGGAGGGACCAA  AATAGGGGCAGACGGAA  TGTGGTACTGAGGGAACGT  GCTCCGCAAAGCAAAGA  CAAAAGGAAGGGGACGTATA |
| β-actin RTF | CAGCCTTCCTTCCTGGGTATGG |
| β-actin RTR | GAGGGAGCGAGGGCAGTGATT |
| vp28 RTF | AGCTCCAACACCTCCTCCTTCA |
| vp28 RTR | TTACTCGGTCTCAGTGCCAGA |
| **RNAi**  Chi2iF  Chi2iR  Chi13iF  Chi13iR | GCGTAATACGACTCACTATAGGCGGCAAATACCGACCTGA  GCGTAATACGACTCACTATAGGTGAAAGCGTAGTGAACCTCC  GCGTAATACGACTCACTATAGGCTGCGAGAAACAGACCACA GCGTAATACGACTCACTATAGGTTAGCGAGCAGCCAAAGAA |
| EcRiF | GCGTAATACGACTCACTATAGGTGGAATAACTGACGACGACT |
| EcRiR | GCGTAATACGACTCACTATAGGTTTGTTTGCCACATACGC |
| RxRiF | GCGTAATACGACTCACTATAGGCCCCTCCAGTTACTCCAA |
| RxRiR | GCGTAATACGACTCACTATAGGCCTCTTTATCTCCCTTCGTC |
| FTZiF | GCGTAATACGACTCACTATAGGCCATCTACATCAACGCATACA |
| FTZiR | GCGTAATACGACTCACTATAGGAGCATCTCCATTAGCAAGG |
| E75iF | GCGTAATACGACTCACTATAGGGAGGTGCTCCTGGTGCGTCT |
| E75iR | GCGTAATACGACTCACTATAGGGCTGCGGTGTGCTCAGTCATC |
| BrciF  BrciR  HR4iF  HR4iR  E93iF  E93iR | GCGTAATACGACTCACTATAGGATGTAACCTTCGCCAACTT  GCGTAATACGACTCACTATAGGGGCTTCACTTAGCACCGT  GCGTAATACGACTCACTATAGGAGACCTCGTCCTGCTCG  GCGTAATACGACTCACTATAGGGGTCAGTGGTGGTCCTAAA  GCGTAATACGACTCACTATAGGCTGTCCTCTTCCCGTTCG  GCGTAATACGACTCACTATAGGCGTTCAATCTGGACTTGCTT |
| GFPiF | GCGTAATACGACTCACTATAGGTGGTCCCAATTCTCGTGGAAC |
| GFPiR | GCGTAATACGACTCACTATAGGCTTGAAGTTGACCTTGATGCC |
| **ChIP** |  |
| Chi2ChIPF | ATTTCCCTTTTCCTGTCCT |
| Chi2ChIPR | TTCCCCCTAACACCCTCCT |
| **EMSA**  Chi2WT2F  Chi2WT2R  Chi2Mut2F  Chi2Mut2R | TGAAGGAAGAGGGGCAGTAAGTAAGTTGGGGCAGG  CCTGCCCCAACTTACTTACTGCCCCTCTTCCTTCA  TGAAGGAAGAGCCACCGTAACTAAGTTGGGGCAGG  CCTGCCCCAACTTAGTTACGGTGGCTCTTCCTTCA |
