# Supplementary material for: Modulation of host lipid metabolism by virus infection leads to exoskeleton damage in shrimp
Source: PLoS Pathog. 2024 May 13;20(5):e1012228. doi: 10.1371/journal.ppat.1012228 (PMC11115362; doi:10.1371/journal.ppat.1012228)
Supplement: Attachment — Submitted filename: Response to comments 0401 0402.docx [file ppat.1012228.s009.docx]

**Part I - Summary**
Please use this section to discuss strengths/weaknesses of study, novelty/significance, general execution and scholarship.

Reviewer #1: In this manuscript, the authors found that white spot syndrome virus (WSSV) infection can alter shrimp lipid metabolism mediated by E75-Chi2-Chitin signaling to damage to the exoskeleton. Some aspects or conclusions should be carefully verified or needs more experimental evidences.

Reviewer #2: In the current manuscript, the authors demonstrated a serial of detailed evidence to show the mechanisms of the loose and softening of exoskeleton in shrimp after WSSV infection and uncovered the relation of chitinase induced exoskeleton hydrolysis with the lipogenesis of LCFAs metabolism via an E75 receptor. Overall, the MS was written well, and the experimental design as well as the results presentation are all very good. It shed the valuable lights on better understanding of the pathogenicity of WSSV, and also provide useful directions for preventing the WSSV-caused disease in shrimp aquaculture industry.

Reviewer #3: The study by Wang et al. sheds light on the molecular mechanism underlying the damage to shrimp exoskeletons following viral infection. The authors demonstrate that WSSV infection reduces chitin levels in the cuticle by upregulating the expression of chitinase Chi2 in the epidermis. They further identify the nuclear receptor E75 as a key player in the WSSV-induced upregulation of Chi2. Moreover, WSSV infection increases host lipid profiles, with enriched saturated long-chain fatty acids (LCFAs) serving as a switch for E75's transcriptional activity. The authors also show that inhibiting LCFA generation improves the mechanical properties of shrimp exoskeletons. By elucidating this intriguing mechanism, the study significantly advances our understanding of WSSV pathogenesis and arthropod-virus interactions. Overall, the study is well-executed, and the results are compelling and robust. These findings hold relevance for a wide readership of PLoS Pathogens. However, certain concerns need to be addressed before publication in this journal.

**R：**We appreciate all comments from three reviewers which were really valuable for us to improve this manuscript. We have addressed the reviewers’ concerns, and the new results indeed strengthened the conclusions of the current study. We hope the revised manuscript could meet the standard for PLoS Pathogens.

**Part II – Major Issues: Key Experiments Required for Acceptance**
Please use this section to detail the key new experiments or modifications of existing experiments that should be absolutely required to validate study conclusions.

Generally, there should be no more than 3 such required experiments or major modifications for a "Major Revision" recommendation. If more than 3 experiments are necessary to validate the study conclusions, then you are encouraged to recommend "Reject".

Reviewer #1: Major aspects:
1. One of the major defects is that all knockdown experiments did not detect protein levels, because that mRNA level knockdown does not represent protein level knockdown. It is necessary to confirm the knockdown efficiency of relevant protein levels, especially chi2 and E75.

**R：**Thanks for your suggestion. We have detected the knockdown efficiency of E75 protein level and shown this data in current Fig. S3. However, since Chi2 is a very big protein with a predicted MW of 353 kD, we failed to make a specific Chi2 antibody. Therefore, we only show the knockdown efficiency of *Chi2* mRNA level. However, the data in Fig 2B that knockdown of *Chi2* expression led to significant decrease in chitinase activity may support that the Chi2 protein level is indeed suppressed after *Chi2* dsRNA application. Thank you.

2. Shrimp motor abilities are key parts of these results, so the authors should provide more intuitive and convincing evidences, for example, behavioral devices and videos or other experimental records. Besides, examine how the data is analyzed and interpreted, particularly the connection between lipid metabolism and WSSV infection in shrimp. The conclusions should be strongly supported by the data.

We have provided the videos recording the shrimp swimming trajectory in current supplemental files. We have added more detailed description of the behavioral test and in the section of Materials and methods (current line 509-517). Thanks for your suggestion.

For the connection between lipid metabolism and WSSV infection, it had been revealed by the colleagues that the manipulation of host lipid metabolism facilities the virus infection. At the early stage of infection, WSSV induces lipolysis to provide material and energy for virus replication; at the late stage of infection, WSSV induces lipogenesis to complete virus morphogenesis by supplying LCFAs (Nr et al., Commun Biol 2023, 6:546; Heish *et al*., Dev Comp Immunol 2015, 1:85-95). In the current study, we proved that the increase in LCFAs led to damage to exoskeleton and impairment of motor ability, and that interfering with the virus-manipulated host lipid metabolism could restore the shrimp cuticle integrity and motor ability. Therefore, based on available knowledge and our present findings, we made the speculation but not a conclusion, in current line 51, 282 and 382, that the manipulated lipid metabolism might contribute to the transmission of WSSV. Thank you.

3. More molecular biological and in vivo experiments are needed to confirm the LCFAs-E75-Chi2-Chitin cascade in shrimp. More importantly, it doesn’t know how the LCFAs-E75-Chi2-Chitin is activated by WSSV infection or can also activated by other pathogens such as DIV1 or bacterial infection, so does this have uniqueness for WSSV or not?

**R：**Thanks for your suggestion. Following your suggestion, we have injected the MA into the shrimp in which the E75 expression was pre-silenced, and detected *Chi2* expression, epidermis chitinase activity and exoskeleton chitin content. As shown in current Fig. 5B-D, E75 knockdown deprived the ability of MA in affecting the chitin metabolism. These data further confirmed the sequential LCFAs-E75-Chi2 cascade. We have added the description of these results in current line 232-241.

Actually, this cascade is an aftereffect of the modulation of host lipid metabolism by WSSV infection. It had been revealed that WSSV infection alter shrimp lipid metabolism by increasing the amount of LCFAs at the late stage of WSSV infection to supply blocks for the production of new intact virions (Ng *et al*., Commun Biol 2023, 6:546). These LCFAs were found as a signal to induce the E75-mediated *Chi2* induction in this study. According to published data, both DIV1 and Vibrio bacterial infection would cause the lipid metabolic reprogramming of shrimp by increasing the amounts of a series of fatty acids, suggesting that the shrimp lipid metabolism might be also altered by other pathogenic infection (He *et al*., Front Immunol 2022, 13:982717; Kumar *et al*., Front Microbiol 2021, 12:631468). In particular, palmitic acid which is concerned in our study was found as a significantly enriched metabolite after the AHPND-causing strain infection. Therefore, the mechanism revealed in this study may be also present after other pathogenic infection. Nevertheless, whether this is the case deserves further study to clarify. We have added some discussion about this issue in the section of Discussion (current line 367-375). Thank you.

4. Why choose the only 72 hpi for testing the mechanical performance of exoskeleton, impaired motor ability and others. How the expression profile of Chi2 and E75 in response to WSSV. Whether the dynamic process of exoskeleton is related to the expression of Chi2 and E75 induced by WSSV.

**R：**Considering that shrimp usually exhibit obvious syndromes at the late stage of WSSV infection, we chose 72 hpi for the testing. Actually, this time point coincides well with the temporal expression profiles of *Chi2*. We had shown the expression profiles at mRNA level of both *Chi2* and *E75* in the original Fig. S2B, and have added the protein level data of E75 in current Fig. S2C. As shown, there is no obvious change of E75 expression after WSSV infection, whereas *Chi2* expression was significantly induced by WSSV infection. The *Chi2* expression showed the highest induction at the late stage of infection (48-72 hpi). Therefore, we believe 72 hpi should be an appropriate time to evaluate the exoskeleton performance. Thank you.

Reviewer #2: NA

Reviewer #3: 1. The authors proposed that the increase of LCFAs led to the enhanced Chi2 transcription by elevating the transcriptional activity of E75. However, E75 expression may also be upregulated by WSSV infection or LCFAs application, and the increase of E75 amount may also lead to the increase of Chi2 transcription. Though the authors have proved that there is no change in E75 mRNA level after WSSV infection (Fig. S2), a protein level data is needed to fully support the current hypothesis. Moreover, the specificity of E75 antibody should also be verified.

**R：**Many thanks for your suggestion. We have provided the protein level data of E75 expression profiles in current Fig. S2C, and the new data confirmed that WSSV infection did not influence the expression of E75. We have also verified the RNAi efficiency at the protein level and have shown this data in current Fig. S3. The decrease of the band intensity after E75 dsRNA application is a good evidence for the specificity of E75 antibody. Thank you.

2. It seems that E75 regulates the expression of Chi2 in an ecdysone-independent manner. Is there any change in the ecdysone concentration after WSV infection? Pathogenic infection usually leads to the variation of ecdysone titer in insects. How about the case in shrimp? I suggest to determine the ecdysone titer to check whether WSSV infection would interfere with the ecdysone system.

**R：**Thanks a lot for your suggestion. Following your suggestion, we have determined the variation of hemolymph ecdysone titer after WSSV infection. As shown in current Fig. S5, WSSV infection indeed led to an increase of ecdysone titer (about 2 folds of induction). However, in the physiological text, only the induction of ecdysone titer with more than 20 folds can drive the molting process. Therefore, we believe that the *Chi2* induction is not due to the slight variation of ecdysone titer after WSSV infection, but due to the LCFAs-E75-Chi2 cascade. We have also added some discussion about this issue in current line 333-336.

3. This study revealed how shrimp exoskeleton is impaired by viral infection. What is the biological significance of this mechanism? Is there any possibility to apply the current finding into the disease control in shrimp culture? The authors can make a deeper discussion on this issue.

**R：**The shrimp exoskeleton consists of multiple joined plates and attaches to muscle, thus allowing for complex movement. We have shown that the damage to the shrimp exoskeleton after WSSV infection would significantly impair the movement of the infected shrimp. As the consumption of dead or feeble WSSV-infected shrimp by healthy shrimp is the major natural transmission route of WSSV, the decrease in moving ability might increase the chance of an infected shrimp being consumed by a healthy shrimp. Therefore, the damage to the shrimp exoskeleton may make a considerable contribution to the natural transmission of WSSV in aquaculture farms.

This study suggests the potential to develop an approach to inhibit the transmission of WSSV in farms by inhibiting the virus-caused abnormal induction of *Chi2*. We have shown that interfering with the WSSV-induced lipogenesis could restore the mechanical performance of shrimp exoskeleton and the shrimp motor ability. Identification of certain chemicals which compete with the LCFAs for binding to E75 may be another alternative to block the induction of *Chi2* and to suppress WSSV transmission. We have added these discussions in current line 279-283 and 292-295. Thank you.

**Part III – Minor Issues: Editorial and Data Presentation Modifications**
Please use this section for editorial suggestions as well as relatively minor modifications of existing data that would enhance clarity.

Reviewer #1: 1. Ensure that the methods used for Chitinase activity assay, RNA interference, Monitoring shrimp motor ability, etc. are clearly described. Detailed methods allow for better reproducibility and understanding of the techniques involved. For Chitinase activity assay, how to set internal parameters to eliminate differences between groups?

**R：**We have enriched the description of these experimental procedures in the section of Materials and methods. The chitinase activity was expressed as the enzyme unit per mg of sub-cuticular tissue protein. We have added this information in current line 477-478. Thank you very much for your suggestion.

2. In many places, the p value should be italic. The manuscript should be polished by an English-native speaker.

**R：**Thanks for your suggestion. We have edited this manuscript by using a professional English-writing service.

Reviewer #2: For the results (Line 190 -201), Fig. 2D, 2E, 2F, 2G and 2h, all should be Fig. 3D, 3E,3F, 3G, AND 3H.

**R：**We are sorry for this carefulness. We have corrected these mistakes. Thank you.

Reviewer #3: 1. Though the authors have provided the primers for all chitinases, the accession number of these sequences should be provided.

**R：**We have provided the accession numbers of all chitinases in current Fig. S1. Thank you.

2. How about the RNAi efficiency for the genes in Fig. 3B? The authors should validate the RNAi-mediated knockdown efficiency, and show the data in Fig. S3.

**R：**We have provided the data showing the RNAi efficiency in current Fig. S3. Thank you.

3. Is the chitin probe self-made or a commercial one? The source of this probe should be provided in detail.

**R：**This probe is a self-made one. We have added the detailed information about this probe in current line 431-435 and 466. Thank you.

4. Fig. 6G, why there is a shift for the green line?

**R：**The shrimp shell collected for test is not completely flat. If there is a slight downwards bend, there may be an extension in the time for the probe to come into contact with the sample, which is reflected in the shift of the spectrum. However, the bend of the shell does not influence the result, as the shell stiffness is expressed as the force under which the probe punctures the shell. Thank you.

5. Fig. S2A, there seems an error when preparing the figure. A symbol is below the panel of actin.

**R：**Thanks a lot for your carefulness. We have edited this error.

6. Some essential information is lacking in the figure legends. The reviewer has to jump back to the section of Materials and methods from time to time to understand at what conditions were the experiments performed. For example, the excitation source of the Raman spectra should be detailed in the figure legends.

**R：**Following your suggestion, we have edited the figure legends by enriching the description. Thank you.

7. This manuscript would benefit from a careful proof reading since a series of grammar errors and typos are present in the text.

We have edited this manuscript by using a professional English-writing service. Thank you.
